# Medical Education and Critical Care Cardiology in the Modern Era

**DOI:** 10.1016/j.jacadv.2025.101804

**Published:** 2025-05-28

**Authors:** Rebecca Garber, Satish Mishra, Adam Mikolajczyk, Amit Goyal, Daniel Ambinder, Catherine Vanchiere

**Affiliations:** a Department of Internal Medicine, University of Illinois, Chicago, Illinois, USA; b CardioNerds, Baltimore, Maryland, USA; c Department of Cardiovascular Medicine, Vanderbilt University Medical Center, Nashville, Tennessee, USA; d Department of Cardiovascular Medicine, UT Southwestern Medical Center, Dallas, Texas, USA; e Veterans Affairs North Texas Health Care System, Dallas, Texas, USA; f Department of Cardiovascular Medicine, University of Maryland, Baltimore, Maryland, USA; g Department of Cardiovascular Medicine, Johns Hopkins Hospital, Baltimore, Maryland, USA; h Department of Cardiovascular Medicine, University of Pennsylvania, Philadelphia, Pennsylvania, USA

**Keywords:** critical care cardiology, FOAMed, medical education

**Figure:**
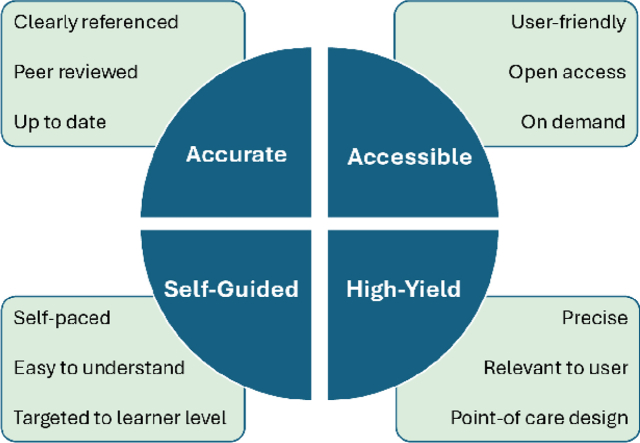

The field of cardiology has evolved significantly since the invention of the stethoscope in the early 19th century by Rene Laennec. From the development of the electrocardiogram to our current pharmacotherapies and advanced mechanical support, our ability to care for critically ill cardiovascular patients has advanced dramatically. While these medical therapies have become crucial to both our patients' care and survival, we would be remiss as a specialty if we overlooked the critical role of medical education in these advancements. A robust understanding of cardiovascular physiology among trainees underpins both future advancements and high-quality patient care. Proper utilization of medical advancements depends on this foundational knowledge. By focusing on the increased complexities of critical care cardiology and the innovations in medical education during this time, we can demonstrate how inextricably linked education is to the field's success.

Medicine has vastly changed over the past 100 years, as evidenced by the birth of critical care cardiology and the evolution of both the pathologies and the advanced therapies offered within the cardiac intensive care unit (CICU). Many sources credit the origins of critical care to Florence Nightingale, a nurse taking care of patients in the 1850s after the Crimean War, as she ensured that the critically ill patients were as close to the nursing staff as possible to be carefully monitored.^1^ A century later in the 1940s to 1960s, the development of mechanical ventilation became a major milestone in response to the polio virus and acute respiratory distress syndrome from the Vietnam War.^1^ The first successful human dialysis treatment occurred in 1945, followed by broader implementation in the 1950s during the Korean War, to combat the rat-borne hantavirus infection that led to kidney failure.^1^ Suddenly, physicians had the ability to offer single-organ life support to their patients and use real-time hemodynamic measurements and lab values to make life-saving adjustments for their critically ill patients. Moreover, the creation of specific units for critically ill patients ensured appropriate monitoring. When President Eisenhower had his heart attack in 1955, he was closely monitored in a hospital for a prolonged period, marking the development of the first-ever coronary care unit (CCU).^2^ Since ∼40% of deaths after heart attacks at that time were due to reversible and preventable fatal arrhythmias, the development of the CCU led to a significant decrease in mortality.^2^ As time progressed and medical interventions improved, critical care units throughout the country were able to care for sicker patients. The CCU, once a place specifically for post-acute coronary syndrome surveillance, transformed into the CICU, a unit capable of offering care for a range of both cardiac and noncardiac issues.^2^ Not surprisingly, multiple studies done in the 1980s to early 2000s found that many of the patients in the CICU were no longer there for just post-ST elevation myocardial infarction care, but instead a multitude of issues, such as cardiogenic shock and heart failure, in addition to other secondary organ failure requiring dialysis or mechanical ventilation.^2^ As the field of critical care cardiology was born, the data began to demonstrate decreased mortality and better outcomes in patients cared for by physicians who were specifically trained in these advanced therapies.^1^^,^^2^

In a similar way, medical education has been through its own evolution over the past century. In the early 1900s, the typical medical school consisted of 2 16-week courses of lectures that students would repeat once before being proclaimed a doctor.^3^ Despite the adoption of germ theory, which revolutionized medicine with the development of antiseptic surgical technique and the discovery of multiple microorganisms that cause disease, the medical curriculum remained unchanged.^3^ The Flexner Report, published by educator Abraham Flexner in 1910, was crucial to the medical education reform by highlighting the deficiencies of medical schools and raising the standards for these schools to produce more uniformly competent doctors. Required prerequisite college courses were identified to ensure all students entering medical school had the appropriate scientific foundations. Additionally, medical school was changed to a 4-year curriculum: the first 2 years became mainly didactic, and the latter 2 focused on research and hands-on clinical training.^3^ The once fully didactic curriculum, which did not reflect the evolving medical landscape, transformed into a more dynamic education by adopting these changes. Students were taught primarily by research faculty at the forefront of medical innovation and became active learners by integrating clinical experience into their education. States subsequently adopted licensing exams, ensuring that all medical graduates performed to a certain standard. Thus, students were now being trained to be problem solvers and critical thinkers rather than perpetrators of outdated medical theory.^3^ Then, after World War II, there was a growing emphasis on developing more specialized training to keep up with the medical advancements of the time.^3^

Since this Flexnerian Revolution of medical education, the challenge for educators—and students—to keep up with the rapid pace of medical discovery and innovation has persisted, further exacerbated by the influx of information with the advent of the internet and artificial intelligence (AI). As Dr William Welch, the first dean of Johns Hopkins School of Medicine, wrote in 1886, “the time has gone by when one mind can encompass all which has been ascertained in the medical sciences.”^3^ If this was the sentiment back then, it rings even truer today. In the 1950s, it was estimated that medical knowledge doubled every 50 years, while only taking 73 days to double in 2020.^4^ Students have gone from being excited to learn everything they possibly can in medical school to being overwhelmed and resigned to learning only what information will be on the test.^3^ While only 7% of internal medicine residents specialized in the early 1960s, 88% specialized in 2021.^4^ Many attribute information overload to this drive toward specialization.^4^

The rapid expansion of medical knowledge demanded new approaches to education. In response to this challenge, the free open-access medical education (FOAMed) movement emerged, empowering the medical community to keep pace with advancements while adapting to the needs of modern learners. The 1990s ushered in a time when medical literature became accessible online, with platforms like PubMed and UpToDate, transforming how information was shared and consumed.^5^ As more people began using the internet, individuals started publishing medical blogs and websites.^5^ Medical resources, such as Life in the Fast Lane, EMCrit, Liver Fellow Network, and Nephrology Fellow Network, were created and became prominent in the medical community.^5^ For the first time, anyone could publish information and learn from and connect with others in different geographical areas.^5^^,^^6^ Soon after, professional societies created their own social media accounts, helping to legitimize the spread of FOAMed in the medical community.^5^^,^^6^ In short, high-quality education was rapidly becoming more readily accessible in ways congruent with the needs of adult learners who tend to be time-constrained and goal-oriented. A busy clinician could now easily access relevant education specific to their immediate clinical questions between seeing patients.

Within cardiology, the CardioNerds platform emerged as a prominent digital education resource aiming to democratize cardiovascular education. What started as a podcast in 2019 to help educate trainees about various cardiovascular diseases developed into a large community during the COVID pandemic.^7^ When quarantining and social distancing became the norm, the CardioNerds were creating connections. Through their Case Report Series highlighting challenging cases from different institutions, social media–based journal clubs and educational “Tweetorials,” Clinical Trials Network, and CardioNerds Academy that teaches members the art of medical education in the digital world, CardioNerds has helped educate learners across the globe about complex and high-level cardiovascular topics.^7^^,^^8^ This accessibility allows the CardioNerds platform to reach learners regardless of the resources available in their own educational setting. Far from oversimplifying complex topics, the platform embraces the intricacies of cardiovascular care, equipping learners with the detailed understanding needed to navigate real-world clinical challenges. Their emphasis on referencing primary literature, pathways for internal peer review, engaging with renowned faculty experts, and collaborating with authoritative professional societies has helped maintain stringent data-driven educational content.

The same milieu of increasing medical complexity creating educational gaps also gave rise to “When the Beat Drops,” an online, peer-reviewed resource created to serve as a resident's guide to the CICU.^9^ Intended to help fill the gap between what residents may have learned in medical school and the advanced pathologies and therapies of their patients in the CICU, the purpose of this website is to make caring for these patients less overwhelming. While there are resources available that review high-yield cardiology topics, many times those resources are too advanced for the resident trainee. Created by residents who are self-aware of the knowledge gaps and peer-reviewed by cardiology fellows, pharmacists, and attendings, “When the Beat Drops” focuses on high-yield, readily accessible information so that trainees can quickly understand the pathologies in front of them at a level appropriate to the learner. From hand-drawn illustrations to the practical information on background, diagnosis, and management, this website equips learners with practical information, links to guidelines, practice-changing trials, and further resources to learn more. The availability of peer-reviewed and trusted resources for trainees like “When the Beat Drops” allows trainees the independence to learn topics on their own and at their own pace. The combination of accurate, accessible, self-guided, and high-yield resources is crucial for modern medical education resources (Figure 1).

**Figure 1:**
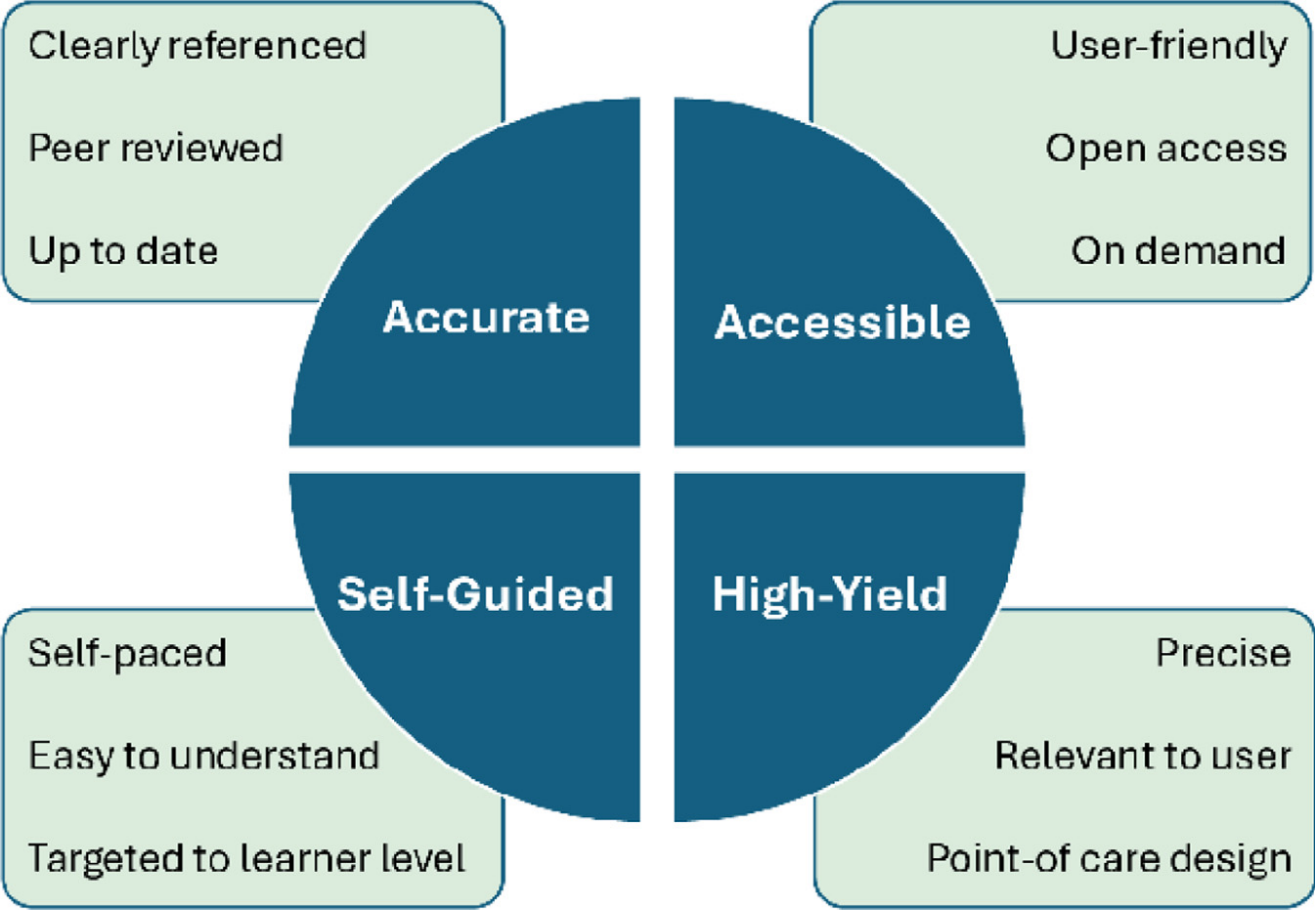
Best Practices for Modern Medical Education Resources This figure highlights the key elements that medical education resources must embody to effectively support learners. Resources should be accurate, ensuring they are clearly referenced, peer-reviewed, and up-to-date to provide trustworthy information. They must be accessible, offering user-friendly platforms that are open access and available on demand, enabling learners to access content anytime. Self-guided features are essential, allowing learners to progress at their own pace with content that is easy to understand and tailored to their level. Finally, resources should be high-yield, focusing on precise relevant information that supports learners' immediate needs, with a point-of-care design to maximize resource utility.

With modern innovations in medical education comes the challenge of navigating emerging technologies like AI. While AI offers remarkable capabilities—synthesizing vast amounts of data, identifying trends, and reducing cognitive burden—it also presents significant risks, particularly around misinformation and overreliance.^4^^,^^10^ AI has access to all free data on the internet; however, it cannot distinguish if the information is reputable or correct, and it cannot access journal articles that require a subscription.^4^^,^^10^ Moreover, when AI cannot find a particular answer, it will “hallucinate” or make up the information and present it as fact.^10^ To the unaware trainee, this can be extremely dangerous as they do not yet have the knowledge base to recognize what is or is not correct. As access to information grows at an astounding rate, it is crucial not to mistake availability of information as true comprehension. This challenge underscores the importance of reliable, peer-reviewed FOAMed resources, which not only equip trainees with accurate information but also reinforce critical thinking skills.

How will we keep up with medical advancements while ensuring we educate our trainees effectively and provide exemplary patient care? There is a great need for the development of curriculum within critical care cardiology to help guide clinicians and trainees on how to appropriately utilize these tools to complement and not supplant one's learning. To advance critical care cardiology, we must innovate medical education alongside technological advancements. Reliable FOAMed resources and thoughtful integration of AI will equip trainees to navigate an ever-growing medical landscape while retaining essential clinical acumen. While the capabilities of AI are infinite, it is still in its infancy, and we are slowly learning how to best incorporate AI within medicine. By continuing to use and develop reliable and easy-to-use FOAMed resources, we help ensure our trainees have the medical knowledge to use and fall back on while also maintaining accuracy in a time when medical information is growing exponentially.

## Funding support and author disclosures

Dr Garber is the creator of the nonprofit, “When the Beat Drops” website. Drs Goyal and Ambinder hold equity in CardioNerds. All other authors have reported that they have no relationships relevant to the contents of this paper to disclose.
